# AI-powered optical coherence tomography shows similar performance to intravascular ultrasound for plaque burden quantification: head-to-head benchmark study against histopathology of native coronary arteries

**DOI:** 10.1093/ehjdh/ztag163

**Published:** 2026-10-12

**Authors:** Jiayue Huang, Ruobing Dai, Xu Yu, Miao Chu, Su Zhang, Simone Fezzi, Sean Hynes, Yining Wang, William Wijns, Shengxian Tu

**Affiliations:** Biomedical Instrument Institute, School of Biomedical Engineering, Shanghai Jiao Tong University, Shanghai 200030, China; Biomedical Instrument Institute, School of Biomedical Engineering, Shanghai Jiao Tong University, Shanghai 200030, China; School of Biomedical Engineering, Southern Medical University, Guangzhou 510515, Guangdong, China; Biomedical Instrument Institute, School of Biomedical Engineering, Shanghai Jiao Tong University, Shanghai 200030, China; Biomedical Instrument Institute, School of Biomedical Engineering, Shanghai Jiao Tong University, Shanghai 200030, China; Division of Cardiology, Department of Medicine, Verona University Hospital, Verona 37126, Italy; Department of Histopathology, University Hospital Galway and University of Galway, Galway H91 YR71, Ireland; Department of Radiology, State Key Laboratory of Complex Severe and Rare Diseases, Peking Union Medical College Hospital, Chinese Academy of Medical Sciences and Peking Union Medical College, Beijing 100730, China; The Lambe Institute for Translational Medicine, Smart Sensors Laboratory and Curam, University of Galway, H91 TK33 Galway, Ireland; Biomedical Instrument Institute, School of Biomedical Engineering, Shanghai Jiao Tong University, Shanghai 200030, China; Department of Cardiology, Erasmus University Medical Center, Rotterdam 3015 GD, the Netherlands

**Keywords:** Optical coherence tomography, Intravascular ultrasound, Histopathology, PCI planning, Artificial intelligence

## Abstract

**Aims:**

Optical coherence tomography (OCT) provides excellent resolution at the cost of shallow penetration, limiting its ability for plaque burden (PB) assessment. A deep learning (DL) algorithm was therefore developed to provide fully automated assessment of PB and vessel size from OCT. We aimed to perform a head-to-head comparison of AI-powered OCT vs. intravascular ultrasound (IVUS) for PB quantification, using histopathology as the reference standard.

**Methods and results:**

Ten post-mortem human hearts were harvested. All 29 major epicardial coronary arteries were perfused and underwent OCT and IVUS pullbacks. After imaging, the arteries were serially sectioned at 1-mm intervals for histopathology. Lumen and vessel contours were derived fully automatically from OCT using the DL algorithm and semi-automatically from IVUS. Histopathological measurements were obtained by experienced analysts. Statistical analyses accounted for within-patient clustering. A total of 852 paired OCT-IVUS histopathology cross-sections were obtained. AI-powered OCT showed strong correlations with histopathology for the assessment of lumen area (*r* = 0.89 [95% CI: 0.88–0.91]), vessel area (*r* = 0.85 [95% CI: 0.83–0.87]), and PB (*r* = 0.76 [95% CI: 0.73–0.79]). The corresponding correlations between IVUS and histopathology were 0.90 (95% CI: 0.89–0.92), 0.86 (95% CI: 0.84–0.88), and 0.79 (95% CI: 0.76–0.81), respectively. Comparisons of the OCT–histopathology and IVUS–histopathology correlation coefficients yielded *P* = 0.336 for lumen area, *P* = 0.444 for vessel area, and *P* = 0.161 for PB. Both OCT and IVUS showed good diagnostic performance in identifying PB > 65%, with the area under the receiver operating characteristic curve being 0.92 (95% CI: 0.90–0.94) and 0.92 (95% CI: 0.88–0.95), respectively.

**Conclusion:**

In this histopathology validation study of 10 post-mortem human hearts, AI-powered OCT enabled fully automated plaque burden quantification and correlated strongly with histopathology, with no statistically significant difference between the OCT–histopathology and IVUS–histopathology correlations.

## Graphical Abstract

**Graphical Abstract:**
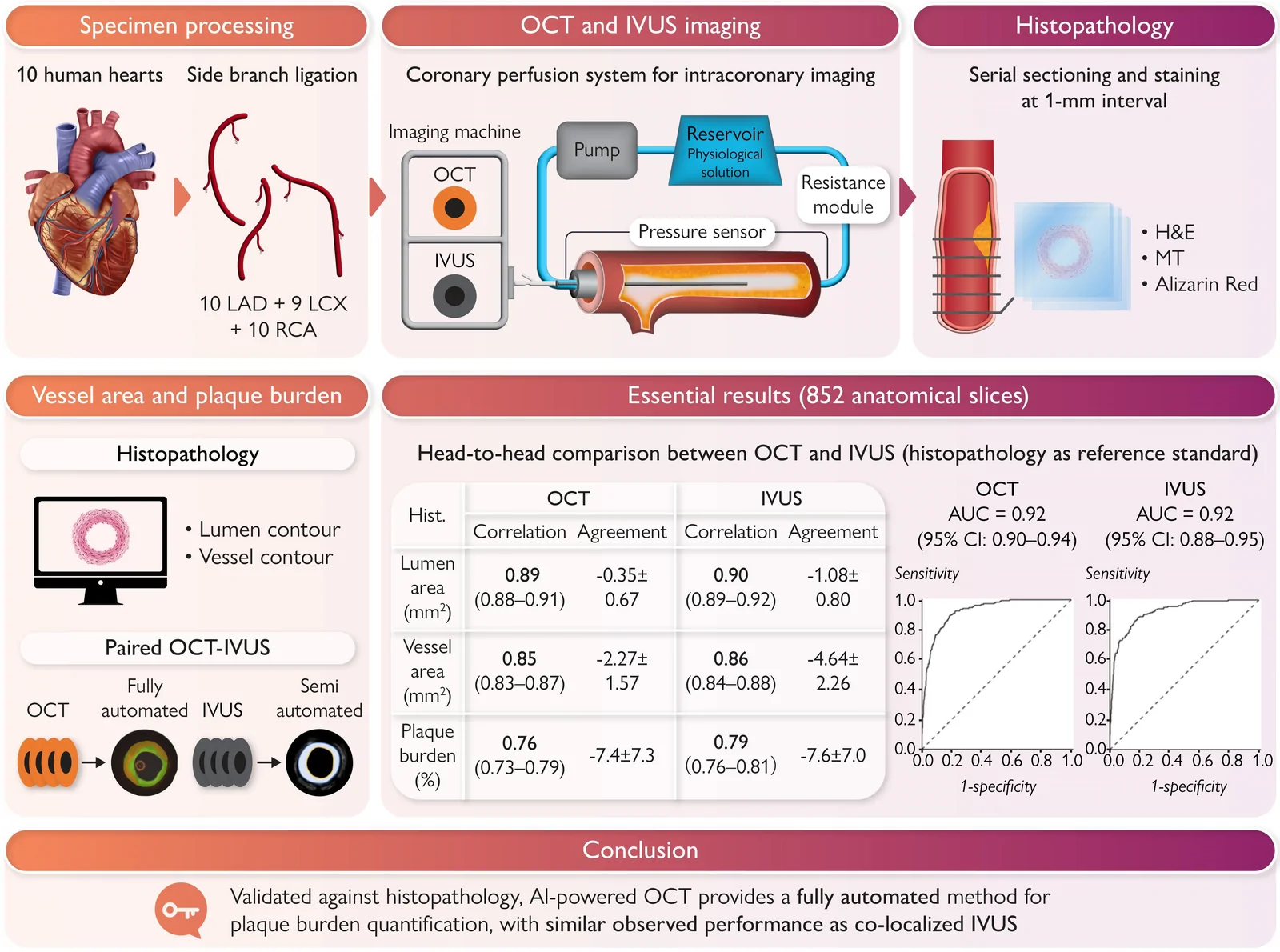

## Introduction

Atherosclerotic plaque remains one of the key drivers of adverse cardiovascular events. Optical coherence tomography (OCT) provides excellent spatial resolution for assessing plaque morphology. However, its limited penetration depth is widely recognized as a potential drawback of this modality, as it may hinder complete visualization of the vessel wall, particularly in the presence of lipid-rich atherosclerotic plaques. This limitation restricts the use of OCT for plaque burden (PB) assessment. PB quantifies the extent to which the vessel is encroached by the atherosclerotic plaque and is a well-established marker of future cardiovascular risk^1–7^ Compared to OCT, intravascular ultrasound (IVUS) provides better tissue penetration, enabling more consistent visualization of the vessel wall, and has therefore been more widely applied for PB assessment.

To address the limitations of OCT, a deep learning (DL) algorithm was developed.^8^ By leveraging contextual information from adjacent OCT cross-sections together with prior anatomical knowledge of vessel morphology, the algorithm is able to provide a reasonable estimation of the vessel contour even when direct visualization is incomplete.^9^ The value of this DL algorithm has previously been validated against pathology from animal models, as well as the co-localized IVUS in patients after bioresorbable scaffold absorption, supporting the concept that AI-powered OCT may extend the utility of conventional OCT.^9,10^ Nevertheless, prior human validation was performed in coronary segments with previous bioresorbable vascular scaffold implantation. Even after the complete resorption of the scaffolds, these segments do not represent truly *de novo* native coronary vessels. The evidence remains limited for validation against native human coronary histopathology, which has been regarded as the reference standard for plaque quantification and is thus essential to confirm the biological fidelity of OCT-derived PB. This study aimed to perform the first head-to-head comparison of AI-powered OCT vs. IVUS for PB quantification, using human coronary histopathology as the reference standard.

## Methods

### Specimens

A total of 10 human hearts were harvested from six males and four females. The cause of death is presented in *Table 1*. Each heart was pressure fixed in formalin for at least 48 h to preserve the *in situ*, perfused geometry of the coronary artery. All three major coronary arteries were then excised from each fixed cadaver heart and ended where the lumen diameter was insufficiently large to permit excision with simple surgical tools (around 1 mm). Stented vessels were excluded as the presence of a stent precludes reliable sectioning and uniform histologic assessment. Vessels that were disrupted during dissection were also excluded.

**Table 1:** Clinal characteristics at time of death

Characteristics	*n* = 10
**Demographic profile**	
Male	6 (60.0%)
Female	4 (40.0%)
Age, years	83 ± 11
**Cause of death**	
Sudden death	1 (10.0%)
Organ failure	2 (20.0%)
Respiratory failure	1 (10.0%)
Lower gastrointestinal bleeding	2 (20.0%)
Pneumonia	1 (10.0%)
Rectal cancer	1 (10.0%)
Myocardial infarction with renal failure	1 (10.0%)
Progressive supranuclear palsy	1 (10.0%)
**Medical history**	
Hypertension^a^	5 (55.6%)
Diabetes mellitus^a^	1 (11.1%)
Hypercholesterolaemia^a^	1 (11.1%)

^a^Data from one post-mortem case was not included due to incomplete medical history, as the family member was emotionally distressed.

This *ex vivo* experimental investigation was approved by the Institutional Review Board of Peking Union Medical College Hospital (Approval No. I-24PJ0053). Written informed consent for donation and research use was obtained from donors prior to death and/or from their legally authorized representative. Details regarding donor heart procurement are provided in the Supplementary Methods.

### Coronary perfusion system and intracoronary imaging

The harvested fixed coronary arteries were ligated at side branches to prevent leakage during perfusion. Both ends of the artery were secured to barbed connectors and mounted in a dedicated coronary perfusion system (*Graphical Abstract*). After achieving stable perfusion, OCT and IVUS pullbacks were acquired according to the manufacturers’ protocols. Further details of the perfusion system and intracoronary imaging procedure are provided in the Supplementary Methods.

### Histopathology

After intracoronary imaging, the arteries were processed for histopathology. Arteries with substantial calcification underwent decalcification with Ethylenediaminetetraacetic acid before being sent for histopathology. Each coronary artery was divided into 1-cm-long subsegments along its course. Each subsegment was then individually oriented during paraffin embedding so that the sectioning plane was perpendicular to its local longitudinal axis. In every 1-mm interval, a series of adjacent 4-μm-thick sections were cut and systematically stained with hematoxylin-eosin (H&E), Masson trichrome, Alizarin red, and CD68 (*Graphical Abstract*).

### Co-registration of histology and intracoronary image

Each histopathologic cross-section was screened for technical adequacy. Sections exhibiting significant tearing, folding, deformation, or tissue loss were not enrolled for co-registration. The matching between histopathologic and intracoronary cross-sections was performed according to their longitudinal position along the artery, determined from the sequential order of the sections and distance from the barbed connector. Side branches served as the principal anatomical landmarks, while luminal configuration and plaque morphology were used as supportive information when needed. For the reproducibility analysis, co-registration was repeated in three randomly selected coronary arteries by the original analyst after an interval of at least 4 months to assess intra-observer reproducibility and independently by a second analyst to assess inter-observer reproducibility. Reproducibility was quantified by the absolute longitudinal difference between the selected OCT frames.

### Histopathology annotation

Quantitative morphometry was performed by experienced analysts using QuPath (version 0.5.1).^11^ The lumen and internal elastic lamina (IEL) were delineated, and the areas enclosed by these contours were defined as lumen area and vessel area, respectively. Cross-sections with side branch ostium preventing reliable contour annotation were excluded from analysis. To investigate the potential impact of lipid accumulation on the performance of the OCT DL, given their propensity to attenuate the optical signal, the lipid pool and necrotic core within the intima were delineated as separate polygon regions of interest, and lipid burden was calculated as the percentage of plaque area occupied by the lipid pool plus necrotic core.

### OCT and IVUS analysis

The PB was fully automatically quantified from OCT pullbacks using a software package (OctPlus, Pulse Medical, Shanghai), in which the DL algorithm was implemented. No manual correction was performed to the OCT analysis. The software automatically delineated the lumen and IEL contours for quantifying lumen area and vessel area, and the tissue in between was classified as fibrotic tissue, lipidic plaque, calcified plaque, macrophage, or cholesterol crystal. A representative example of the software interface is shown in Supplementary material online, *Figure S1*. The DL algorithm uses consecutive OCT cross-sections as pseudo-3D input to incorporate longitudinal contextual information and generate segmentation of the central frame.^8^ For OCT cross-sections with invisible vessel wall, the algorithm estimates vessel geometry by integrating the spatial continuity of vessel-wall information across adjacent cross-sections with learned morphological characteristics of the vessel contour, including its generally regular and approximately circular or elliptical geometry. The histological slides in this study were not used to train or fine-tune the algorithm, thereby preserving their role as an independent reference standard for the current study. Further architectural details are provided in Supplementary Methods.

IVUS cross-sections were analysed semi-automatically using the IvusPlus software (Pulse Medical, Shanghai) at the CardHemo core lab by another analyst (X. Y) who is blinded to the OCT and histology analysis data. A more detailed description of the IVUS model and analysis is provided in Supplementary Methods. The lumen area and vessel area derived from both modalities were recorded and compared with histopathology reference standard. The lumen and vessel diameter were calculated from the lumen and vessel area by assuming the lumen and vessel have a circular shape. PB was calculated as [(vessel area − lumen area)/vessel area] × 100%.

### Statistical analysis

Continuous variables are summarized as mean ± standard deviation (SD) or median (25th-75th percentiles), as appropriate. Categorical variables are reported as counts and percentages. The correlation between histopathology-derived and OCT-derived measurements was assessed using Spearman’s rank correlation, accounting for within-patient clustering. The OCT–histopathology and IVUS–histopathology correlation coefficients, derived from the same co-registered cross-sections, were compared to assess differences in the strength of association with the common histopathological reference. Agreement was assessed using Bland–Altman analysis with a mixed-effects model to account for within-patient clustering. Diagnostic performance of OCT DL and IVUS was evaluated using receiver-operating characteristic analysis, with the area under the curve (AUC) and its 95% confidence interval (CI) estimated by patient-cluster bootstrap resampling with 2000 replications to account for within-patient clustering; the optimal operating threshold was determined by the Youden index. The diagnostic accuracy of OCT-derived and IVUS-derived PB was defined as its classification concordance with histopathology-derived PB, using the same cut-off value of 65% adopted in the most recent studies.^7,9,12^ Sensitivity analyses were additionally performed across PB thresholds ranging from 55% to 70%.^1–7^ All analyses were performed using R (R Foundation for Statistical Computing, Vienna, Austria). A two-sided *P* value <0.05 was considered statistically significant.

## Results

### Study population and baseline characteristics

Ten post-mortem human hearts were obtained, yielding 29 major epicardial coronary arteries (10 LAD, 9 LCX, 10 RCA) for intracoronary imaging and histologic examination (*Graphical Abstract*). Clinical characteristics at time of death were presented in *Table 1*. Among 1122 candidate histological cross-sections, 191 (17.0%) were excluded because tearing, folding, deformation, or tissue loss precluded reliable morphometric assessment. The remaining 931 sections were co-registered with the corresponding OCT and IVUS images. Of those, 79 were excluded due to the presence of side branch ostium preventing reliable lumen and media contour annotation in histology (*Figure 1*), leading to 852 paired OCT-IVUS-histopathology cross-sections for comparison. *Figure 2* and *Figure 3* show 2 representative examples of PB analysis for cross-sections with lipid-rich plaque and calcification which preclude the complete visualization of media in OCT.

**Figure 1:**
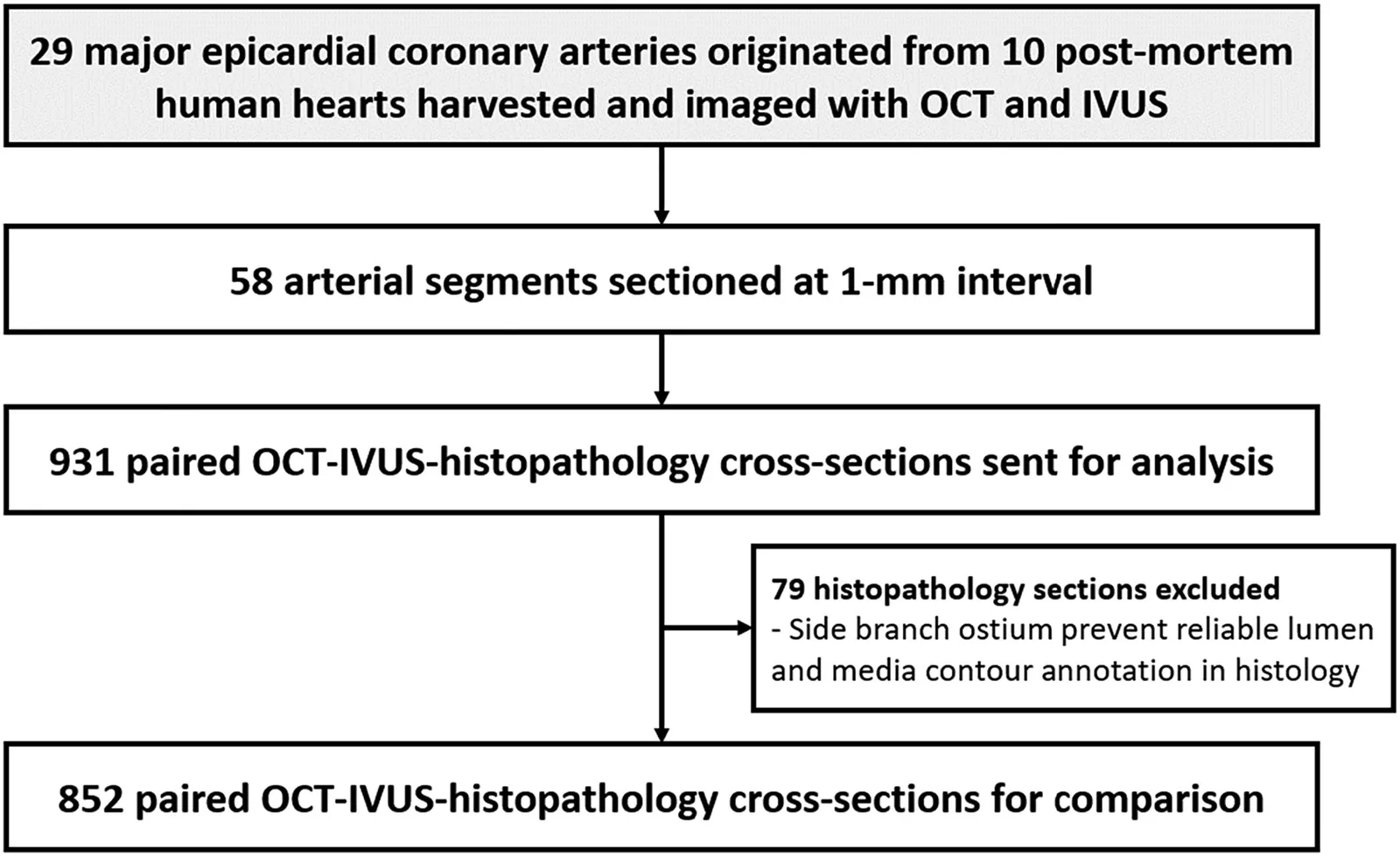
Study flow chart. IVUS, intravascular ultrasound; OCT, optical coherence tomography.

**Figure 2:**
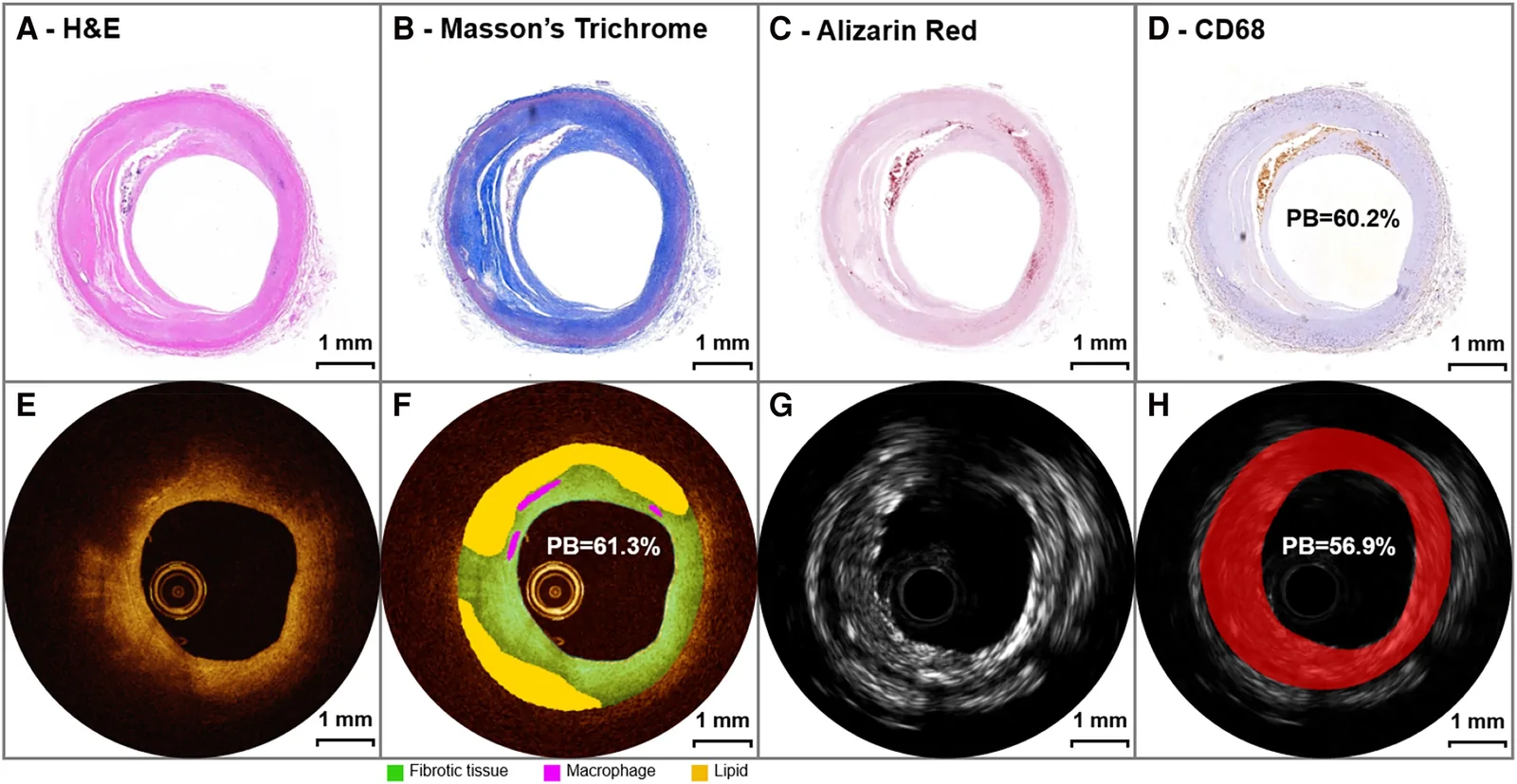
Representative co-registered histopathology, OCT, and IVUS cross-sections of a lipid-rich plaque. Serial histopathological sections are shown with (*A*) H&E staining, (*B*) Masson’s trichrome staining, (*C*) Alizarin red staining, and (*D*) CD68 immunohistochemistry. The corresponding intracoronary images include (*E*) the original OCT cross-section, (*F*) the DL-derived OCT tissue characterization and PB quantification, (*G*) the original IVUS cross-section, and (*H*) the IVUS-derived PB quantification. PB derived from histopathology, OCT, and IVUS are displayed in D, F, and H, respectively. H&E, hematoxylin and eosin; IVUS, intravascular ultrasound; OCT, optical coherence tomography; PB, plaque burden.

**Figure 3:**
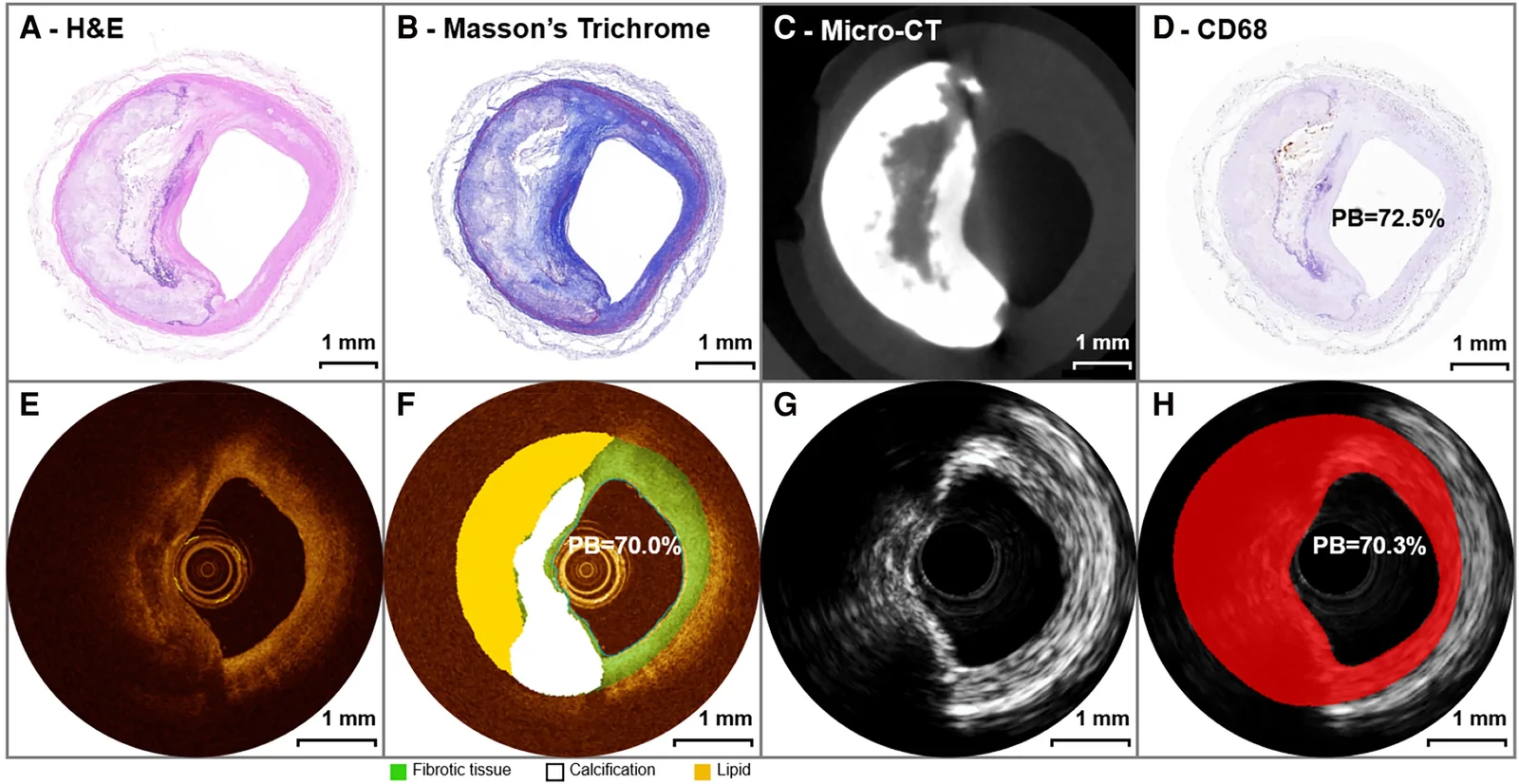
Representative co-registered histopathology, micro-CT, OCT, and IVUS cross-sections of a calcified plaque with lipid-rich tissue. Serial histopathological and micro-CT images are shown with (*A*) H&E staining, (*B*) Masson’s trichrome staining, (*C*) micro-CT, and (*D*) CD68 immunohistochemistry. The corresponding intracoronary images include (*E*) the original OCT cross-section, (*F*) the DL-derived OCT tissue characterization and PB quantification, (*G*) the original IVUS cross-section, and (*H*) the IVUS-derived PB quantification. PB derived from histopathology, OCT, and IVUS are displayed in D, F, and H, respectively. CT, computed tomography; H&E, hematoxylin and eosin; OCT, optical coherence tomography; PB, plaque burden.

### Co-registration reproducibility

Co-registration reproducibility was assessed in 159 histological cross-sections from three randomly selected coronary arteries. For intra-observer reproducibility, the mean absolute longitudinal differences between the selected OCT frames were 0.07 ± 0.09 mm (95% CI: 0.06–0.09), with a corresponding mean absolute difference in OCT-derived PB of 0.81 ± 1.33% (95% CI: 0.61–1.02). For inter-observer reproducibility, the respective differences were 0.22 ± 0.35 mm (95% CI: 0.16–0.27) and 1.12 ± 2.13% (95% CI: 0.78–1.45).

### Lumen and vessel area measurements

*Table 2* summarizes the lumen area, vessel area, and PB as derived from histopathology, OCT DL, and IVUS. The correlation and agreement vs. histopathology-derived area measurements are shown in *Figure 4* and Supplementary material online, *Figure S3*. OCT DL showed strong correlation with histopathology for the assessment of lumen area (*r* = 0.89 [95% CI: 0.88–0.91], *P* < 0.001) and vessel area (*r* = 0.85 [95% CI: 0.83–0.87], *P* < 0.001). The correlation between IVUS and histopathology was 0.90 (95% CI: 0.89–0.92, *P* < 0.001) for lumen area and 0.86 (95% CI: 0.84–0.88, *P* < 0.001) for vessel area, respectively. IVUS-derived measurements showed similar observed correlations with histopathology as OCT-DL, with *P* = 0.336 for lumen area and *P* = 0.444 for vessel area. Both OCT and IVUS exhibited systematic bias compared with histopathology, overestimating lumen area (histopathology—OCT = −0.35 ± 0.67 mm^2^, *P* < 0.001; histopathology—IVUS = −1.08 ± 0.80 mm^2^, *P* < 0.001) and vessel area (histopathology—OCT = −2.27 ± 1.57 mm^2^, *P* = 0.041; histopathology—IVUS = −4.64 ± 2.26 mm^2^, *P* < 0.001).

**Figure 4:**
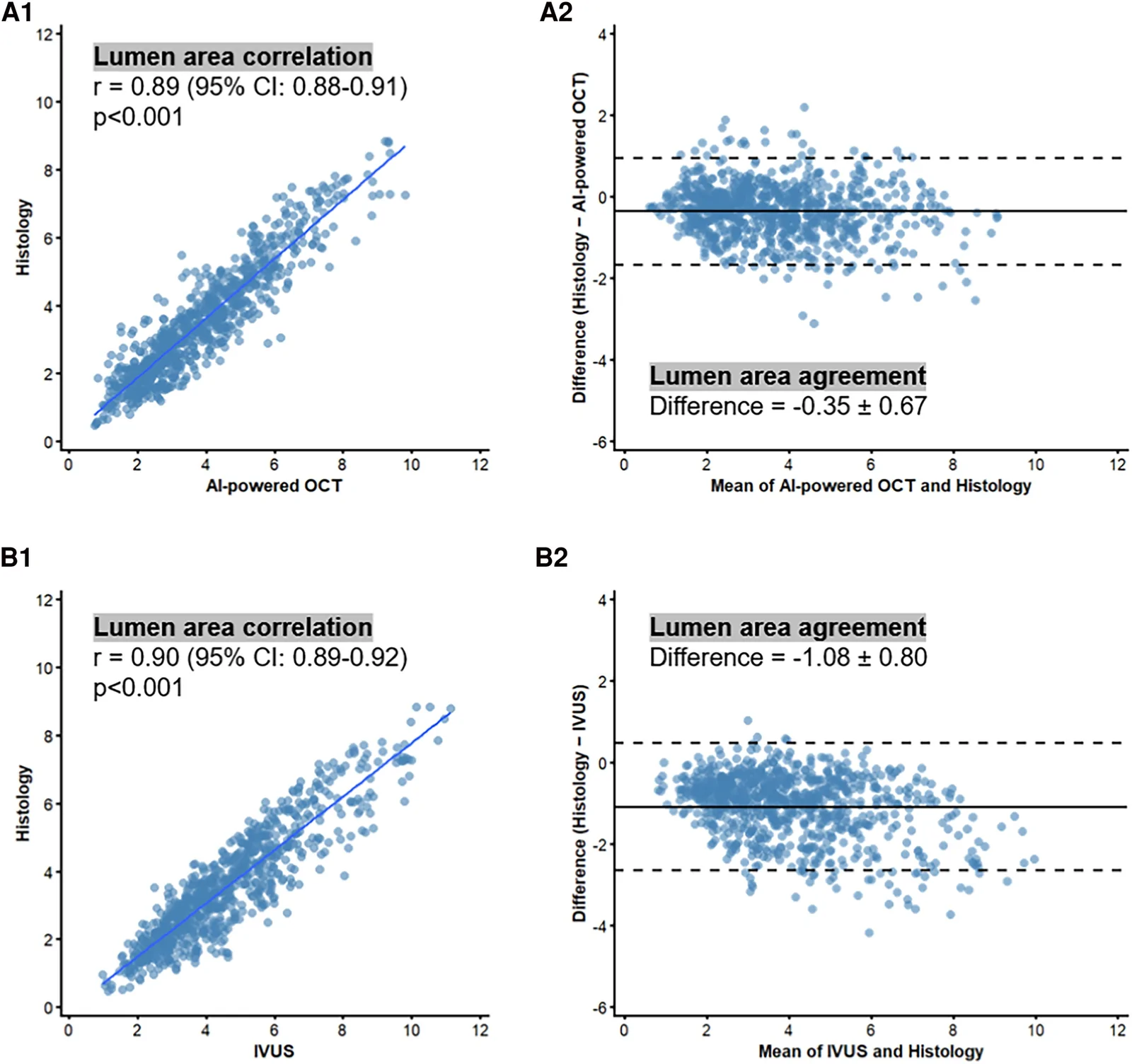
Correlation and agreement between histopathology-derived lumen area vs. OCT DL-derived lumen area (A1-A2) and IVUS-derived lumen area (B1-B2). DL, deep learning; OCT, optical coherence tomography.

**Table 2:** Lumen, vessel, and plaque burden measurements derived from histopathology, OCT, and IVUS

	Histopathology	OCT DL	IVUS
**Lumen area (mm^2^)**	3.25 [2.20, 4.51]	3.58 [2.51, 4.96]	4.23 [3.07, 5.63]
**Vessel area (mm^2^)**	8.00 [5.66, 9.98]	10.24 [8.03, 12.29]	12.29 [9.40, 15.26]
**Plaque burden (%)**	55.7 ± 12.1	63.1 [56.2, 69.4]	63.5 ± 9.02

Variables are presented as mean ± SD when normally distributed and as median [25th–75th percentiles] when non-normally distributed.

DL, deep learning; IVUS, intravascular ultrasound; OCT, optical coherence tomography.

### PB measurements

OCT DL–derived PB showed good correlation (*r* = 0.76 [95% CI: 0.73–0.79], *P* < 0.001) and agreement (histopathology—OCT = −7.4 ± 7.3%, *P* < 0.001) with the histopathology reference (*Figure 5*). IVUS-derived PB demonstrated a similar observed correlation with histopathology as OCT DL did (*P* = 0.161), with the correlation coefficient being 0.79 (95% CI: 0.76–0.81, *P* < 0.001) and the difference being −7.6 ± 7.0% (*P* < 0.001). OCT demonstrated excellent diagnostic performance for identifying histopathology-derived PB > 65% (AUC = 0.92 [95% CI: 0.90–0.94]) (*Figure 6A*), with an optimal Youden-derived threshold of 67.2% (95% CI: 65.6–69.7%). At this threshold, the deep-learning model achieved an overall accuracy of 83%, yielding 169 true positives, 541 true negatives, 122 false positives, and 20 false negatives. IVUS showed similar diagnostic performance (AUC=0.92 [95% CI: 0.88–0.95]) as OCT DL (*P* = 0.881) (*Figure 6B*). The optimal Youden-derived threshold for IVUS was 67.3% (95% CI: 64.8–70.8%). Given that multiple prior studies have applied PB thresholds ranging from 55% to 70%,^1–7^ sensitivity analyses were additionally performed using reference thresholds of 55%, 60%, and 70%. Across these definitions, OCT DL performance remained robust, with AUCs ranging from 0.91 to 0.92 (*Table 3*).

**Figure 5:**
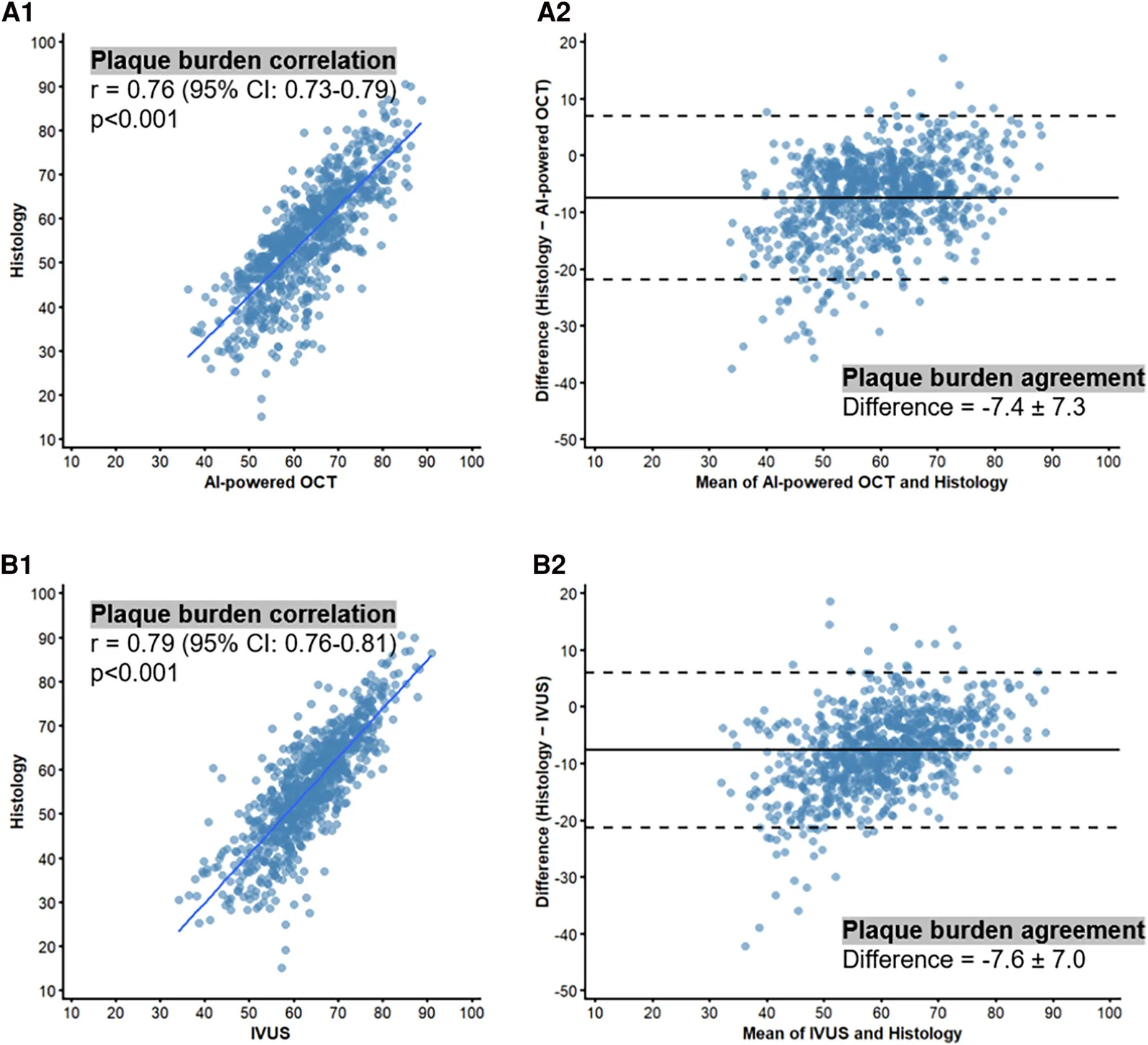
Correlation and agreement between histopathology-derived and OCT DL-derived plaque burden (A1-A2) and IVUS-derived plaque burden (B1-B2) AI , artificial intelligence; IVUS, intravascular ultrasound; OCT, optical coherence tomography.

**Figure 6:**
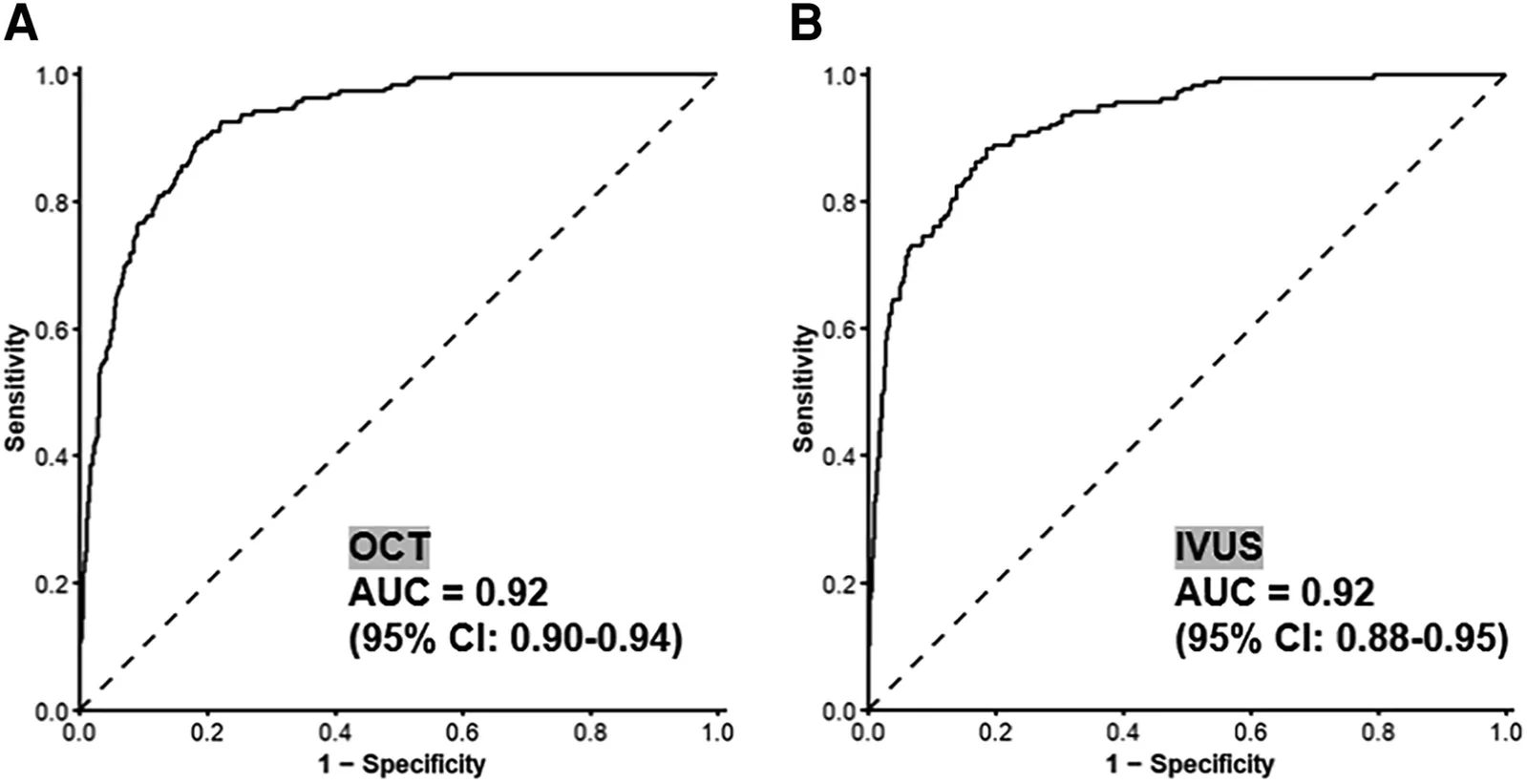
Diagnostic performance of OCT DL-derived (*A*) and IVUS-derived (*B*) plaque burden. AUC, area under the receiver operating characteristic curve; CI, confidence interval; IVUS, intravascular ultrasound; OCT, optical coherence tomography.

**Table 3:** Sensitivity analysis across different histopathology PB thresholds

PB cut-off	OCT		IVUS	
	AUC	Youden threshold	AUC	Youden threshold
**55%**	0.91 95% CI: 0.85–0.94	63.2 95% CI: 58.5–66.3	0.90 95% CI: 0.84–0.94	63.1 95% CI: 61.8–65.9
**60%**	0.91 95% CI: 0.87–0.95	63.6 95% CI: 61.5–67.5	0.90 95% CI: 0.85–0.94	66.3 95% CI: 63.3–67.2
**65%**	0.92 95% CI: 0.90–0.94	67.2 95% CI: 65.6–69.7	0.92 95% CI: 0.88–0.95	67.3 95% CI: 64.8–70.8
**70%**	0.91 95% CI: 0.87–0.95	69.6 95% CI: 65.6–73.5	0.95 95% CI: 0.92–0.97	71.0 95% CI: 67.7–74.2

AUC, area under the receiver operating characteristic curve; CI, confidence interval; PB, plaque burden.

### Impact of lipid accumulation

The lipid burden correlated weakly with the numerical difference between histopathology-derived and intracoronary image-derived PB, with the correlation coefficient being 0.19 (95% CI: 0.12–0.25, *P* < 0.001) for OCT and 0.17 (95% CI: 0.11–0.24, *P* < 0.001) for IVUS, respectively. In the false-prediction group, where the intracoronary image failed to achieve correct risk stratification, the lipid burden did not show significant difference compared to the right-prediction group for both OCT (1.39 [95% CI: 0.10–2.68]% vs. 1.54 [95% CI: 0.31–2.77]%, *P* = 0.626) and IVUS (1.26 [95% CI: 0.00–2.55]% vs. 1.57 [0.34–2.87]%, *P* = 0.305).

## Discussion

To the best of our knowledge, this study provides the first head-to-head histopathological validation of AI-powered OCT vs. IVUS in human coronary arteries. The main findings are as follows: (1) AI-powered OCT provided similar observed performance to IVUS for PB quantification; (2) lipid accumulation did not materially impair the risk stratification performance of OCT-derived PB.

OCT provides unparalleled image resolution but is fundamentally constrained by limited penetration depth, precluding sometimes the reliable and reproducible tracing of the outer vessel boundary—particularly with the presence of lipid-rich plaques that attenuate the optical signal. This constraint directly affects OCT-based vessel size estimation and, consequently, PB assessment. To better address this challenge, we developed a DL model that can provide a reasonable estimation for the vessel contour by leveraging contextual data from adjacent OCT cross-sections and incorporating prior anatomical knowledge of typical vessel morphology.^8^ The performance of this DL algorithm has been previously validated *in vivo* against co-localized IVUS.^9^ While demonstrating substantial concordance, broader clinical implementation was constrained by the exclusive use of post-resorption scaffold segments. Validation against histopathology in swine models has also been performed but are somehow limited due to the lack of full spectrum of advanced human coronary plaques.

The present study extends the prior work in two key respects: (1) we used human histopathology from native coronary arteries as the definitive reference standard; (2) we minimized selection bias by adopting an all-comers post-mortem design. Majority of the previous post-mortem studies comparing intracoronary imaging with histopathology employed targeted sampling guided by the visible light-aiming beam position,^13–15^ potentially introducing selection bias. The present study, on the other hand, systematically imaged all accessible coronary arteries and performed histopathological validation at 1-mm interval. This non-targeted whole-vessel sampling inevitably included technically challenging sections with substantial tearing, folding, deformation, or tissue loss. Although some degree of non-random exclusion related to plaque composition cannot be completely excluded, this approach substantially reduced preselection at the sampling stage. As a result, the final dataset retained a broad spectrum of disease severity, with PB ranging from 30% to 90%. Crucially, the clustering effects were further adjusted to ensure robust generalizability of our findings.

To minimize geometric discrepancies between intracoronary imaging and histopathology, OCT and IVUS were acquired after formalin fixation and under pressure-controlled perfusion. This design prioritized preservation of vascular geometry and close correspondence with the subsequent histopathological assessment. Formalin fixation may nevertheless alter tissue hydration and protein structure, thereby modifying optical or acoustic properties. Although a previous paired study comparing human coronary arteries before and after fixation found no systematic differences in optical properties, comparable evidence regarding the effects of fixation on IVUS acoustic properties remains lacking.^16^ The design of this study therefore sought to balance the preservation of native imaging characteristics with the maintenance of close geometric correspondence to the histopathological reference, and we observed a systematic offset between histopathology-derived vs. OCT DL and IVUS-derived measurements. Both OCT and IVUS provide larger numerical results compared to histopathology, which is in line with the findings from previous autopsy studies.^17–19^ This systematic discrepancy likely stems from the histopathology process: after sequences of desiccation, paraffin embedding, sectioning, and alteration by glutaraldehyde, the histomorphometry of specimens tends to be slightly altered, causing non-uniform and composition-dependent tissue shrinkage that might introduce the systematic difference between imaging and histology reference.^20^ These technical considerations explain the size-dependent variance observed in our Bland–Altman analysis (*Figure 4* and Supplementary material online, *Figure S3*), where greater discordance occurred in the cross-sections with larger dimensions. In addition, these histological processing-related dimensional changes might also influence the absolute interpretation of the histopathology-derived 65% PB threshold. Nevertheless, the consistent diagnostic performance observed across prespecified histopathology-derived thresholds from 55% to 70% supports the robustness of the principal findings.

### OCT vs. IVUS

The complementary nature of OCT and IVUS has been well-recognized: OCT provides superb image resolution for the assessment of lumen architecture and plaque morphology, while IVUS is favoured for its deep penetration enabling PB quantification. In this study, however, we observed a similar performance of OCT and IVUS for lumen area measurements. It seems that the intrinsic resolution advantage of OCT did not translate into a markedly stronger correlation with histopathology. This finding may be partly explained by the fact that OCT analyses were fully automated while IVUS images were analysed in a semi-automated fashion, allowing manual adjustments in case of ambiguous cross-sections. More importantly, for PB assessment—an application for which OCT has long been considered less suitable—OCT showed good correlation and agreement with histopathology without a meaningful decrement compared to IVUS (*P* = 0.161). It should also be noted that OCT and histopathology used the IEL to define vessel area, whereas IVUS used the external elastic lamina (EEL) in accordance with conventional IVUS methodology; therefore, a small systematic difference related to inclusion of the medial layer in IVUS measurements cannot be excluded. However, the practical impact is likely limited, as the media is relatively thin (typically ∼0.1–0.2 mm) and represents only a small proportion of the measured plaque-plus-media area. Taken together, these findings are encouraging as they support the concept that the intrinsic limitation of OCT—namely, the shallow penetration depth—might be partly compensated with the use of a dedicated DL algorithm. While this should not be interpreted as complete interchangeability, it raises the possibility that, for selected applications focusing on PB assessment, OCT might be sufficient when aided by AI.

### Impact of lipid accumulation

Quantitative analysis showed a statistically significant but modest positive correlations between lipid burden and the signed difference between histopathology-derived and both OCT-derived and IVUS-derived PB. Considering the overestimating tendency of intracoronary imaging,^17–19^ a positive association here indicates that greater lipid burden was associated with a reduced magnitude of overestimation by both modalities. This bias cancellation might partly stem from boundary detection uncertainties at lipid-rich plaques, which introduce a counterbalancing underestimation tendency that partially offsets their baseline overestimation. Notwithstanding this modest numerical influence, the lipid burden was not significantly different between the OCT DL false- and right-prediction groups, indicating that the signal attenuation from lipid was not clearly associated with a higher likelihood of classification error in the present cohort. Nevertheless, this finding should be interpreted with caution, as lipid-rich plaque and necrotic core may be more susceptible to tissue shrinkage during histological processing,^20^ thereby influencing both lipid-burden quantification and histopathology-derived lumen and vessel area measurements, and potentially confounding the observed association between lipid burden and the difference in PB measured by OCT vs. histopathology. Future studies incorporating frozen-section histology with lipid-specific staining may reduce lipid extraction and provide a more direct assessment of this relationship.

### Clinical perspectives

Our study provides the first human histopathology-validated evidence for OCT-derived PB using DL. Importantly, as this is a fully automated algorithm-based approach, it could be efficiently and reproducibly applied either prospectively in cathlab or retrospectively to existing datasets. The good performance of OCT DL for PB quantification, when integrated with OCT-based thin-cap fibroatheroma detection and physiological assessment (such as optical flow ratio),^21^ may enable a more comprehensive evaluation of the target lesion, thereby facilitating a more precise risk stratification.

From a procedural perspective, the ability of OCT-DL to estimate vessel dimensions is also of potential clinical interest. Accurate vessel-size assessment is an important component of stent sizing, but OCT-based sizing strategies have long been regarded as less practical due to its shallow penetration depth. Although several novel stent sizing strategies have been previously proposed for OCT,^22–24^ concerns remained regarding the relatively low feasibility (failed in around 15% of the cases), the requirement for manual measurements, and the presence of frequent and potentially hazardous stent oversizing. The present findings showed that automated OCT-derived vessel-size estimates closely tracked the histopathology-derived reference (r = 0.88), with similar observed performance to IVUS (*P* = 0.444), supporting the potential of this automated approach as a basis for future stent-sizing strategies. Nevertheless, its clinical utility for procedural planning and device selection will require prospective validation in patients undergoing percutaneous coronary intervention.

Meanwhile, this study also revealed how lipid accumulation might affect algorithm performance. The finding that lipid burden did not differ significantly between the right- and false-prediction groups challenges the conventional knowledge that lipid-rich plaque necessarily limits reliable OCT-based PB assessment and supports the application of AI-powered OCT for clinically meaningful risk stratification.

### Study limitations

The present study has several limitations. Firstly, although we mitigated potential vessel collapse by pressure-fixation and controlled perfusion, the histopathology geometry cannot be 100% the same as the OCT and IVUS geometry because of non-uniform and plaque composition-dependent tissue shrinkage during histological processing, the potential influence of residual oblique sectioning, and minor longitudinal mismatch during co-registration. For the same reason, histopathology, although it remains the most direct tissue-based reference currently available, should not be regarded as an entirely unbiased geometric standard. In addition, formalin fixation may differentially affect the optical and acoustic properties of OCT and IVUS, potentially influencing their relative observed performance. Consequently, part of the apparent between-modality difference may reflect fixation-related effects rather than intrinsic differences between the two modalities. The PB thresholds evaluated and derived in this study should also be interpreted with appropriate caution due to the potential impact of tissue shrinkage. Nevertheless, the consistent diagnostic performance across sensitivity analyses using histopathology-derived PB thresholds from 55% to 70% supports the robustness of the principal finding regarding AI-powered PB estimation. Secondly, the cross-sections with side-branch ostium were excluded from comparison as it might impair reliable and reproducible annotation on histopathology; the DL’s performance at the side branch site warrants further investigation. Finally, the donor-derived nature of this study precluded the prospective control over cohort characteristics and anatomical diversity, and an elderly (mean age 83 ± 11 years) donor cohort may overrepresent advanced or calcified disease relative to the broader population undergoing clinical OCT. Although extensive whole-vessel sampling provided broad anatomical and pathological coverage within the cohort, further studies are warranted to confirm the generalizability of these findings across the broader spectrum of human coronary atherosclerosis.

## Conclusions

In this histopathology validation study of 10 post-mortem human hearts, AI-powered OCT enabled fully automated PB quantification and correlated strongly with histopathology, with no statistically significant difference between the OCT–histopathology and IVUS–histopathology correlations.

## Lead author biography


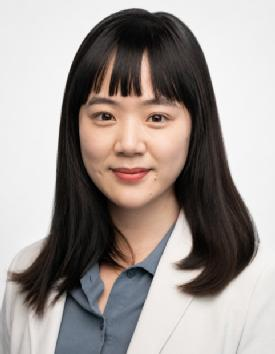
Dr. Jiayue Huang is a cardiovascular researcher at Shanghai Jiao Tong University. She received her PhD under the supervision of Professor William Wijns and Professor Shengxian Tu. Her research focuses on cardiovascular biomechanics, intravascular imaging, and AI-enabled assessment of coronary atherosclerosis. Her current work integrates medical imaging, computational modelling, and human histopathology to improve quantitative plaque characterization and risk stratification, with the aim of advancing translational approaches for identifying high-risk coronary atherosclerosis. She was selected as the winner of the Young Investigator Award in Artificial Intelligence in Cardiology at ESC Congress 2026.

## Supplementary Material

ztag163_Supplementary_Data

## Data Availability

The data underlying this article cannot be shared publicly due to restrictions related to the use and distribution of human post-mortem tissue-derived data. The data will be shared on reasonable request to the corresponding author.
