# Supplementary material for: AI-powered optical coherence tomography shows similar performance to intravascular ultrasound for plaque burden quantification: head-to-head benchmark study against histopathology of native coronary arteries
Source: Eur Heart J Digit Health. 2026 Oct 12;7(9):ztag163. doi: 10.1093/ehjdh/ztag163 (PMC13695069; doi:10.1093/ehjdh/ztag163)
Supplement: Supplementary file 1 [file ztag163_supplementary_data.docx]

**Supplementary Material**

**Table of contents**

**Supplementary Methods	S2**

**Supplementary Figures	S4**

**Supplementary Methods**

***Donor heart procurement***

Donor hearts were obtained through the National Human Brain Bank for Development and Function, Chinese Academy of Medical Sciences and Peking Union Medical College, Beijing, China.

***Coronary perfusion system and intracoronary imaging***

The pump (Model 769 Peristaltic Pump, Computerized Imaging Reference Systems) generated pulsatile flow at 60 cycles/minute, simulating in vivo hemodynamic conditions with pressures ranging from 80 to 120 mmHg. After achieving stable perfusion, a 0.014-inch guidewire was advanced into the artery through a Y connector, followed by introduction of IVUS (OptiCross 40MHz and POLARIS, Boston Scientific) and OCT imaging catheter (C7-XR, St. Jude Medical and Dragonfly Optis, Abbott). Automated pullbacks were performed according to manufacturers’ protocol. For arteries exceeding the effective pullback length, sequential pullbacks were acquired to ensure complete imaging of the target artery.

***OCT deep learning algorithm***

The DL algorithm is based on a U-shaped encoder–decoder convolutional neural network, consisting of a contracting path for hierarchical feature extraction and an expansion path for full-resolution segmentation. Down-sampling and up-sampling operations are combined with batch normalization, and horizontal skip connections together with vertical feature bridges are used to preserve multi-scale spatial information. A stack of consecutive OCT cross-sections is used as pseudo-3D input, enabling the model to incorporate longitudinal contextual information and produce segmentation of the central frame. The algorithm had previously been trained using 10517 expert-annotated cross‐sections from 509 OCT pullbacks.

***Semi-automated IVUS analysis***

The IvusPlus software automatically delineated the lumen and external elastic lamina (EEL) for the quantification of lumen area and vessel area, using a high-resolution deep learning architecture consisting of a High-Resolution Network (HRNet) backbone for robust feature extraction and an Object-Contextual Representation (OCR) head to aggregate contextual information for precise semantic segmentation (**Supplementary Figure 1**). The model was fed with pseudo-3D input by stacking 3 consecutive IVUS cross-sections as separate channels to integrate spatial and temporal information along the longitudinal direction. A hybrid loss function combining multi-class cross-entropy (CE) loss and Dice loss was used to address the class imbalance between the background and the typically narrow, challenging media and intima regions. In case of suboptimal delineation, manual adjustment would be performed by experienced analysts.

**Supplementary Figure 1. Representative software interface of AI-powered OCT analysis.**

A representative OCT pullback analysed by the OctPlus software is shown. The interface displays in the upper panel the cross-sectional view with automated lumen and vessel contour delineation, color-coded tissue characterization, and plaque burden quantification. The longitudinal view in the lower panel provides pullback-level visualisation of the lumen and vessel size along the analysed segment. The 3D reconstruction in the right panel illustrates the spatial distribution of the lumen, vessel boundary, and plaque components throughout the pullback.

Cal. = calcified plaque; Cry. = cholesterol crystal; Fib. = fibrous tissue; Lip. = lipidic plaque; Mac. = macrophage; OCT = optical coherence tomography.

**Supplementary Figure 2. Schematic diagram of the IVUS model architecture**

**Supplementary Figure 3. Correlation and agreement between histopathology-derived vessel area versus OCT DL-derived vessel area (A1-A2) and IVUS-derived vessel area (B1-B2)**

AI = artificial intelligence; IVUS = intravascular ultrasound; OCT = optical coherence tomography.
